# Biodistribution of Mesenchymal Stem/Stromal Cells in a Preclinical Setting

**DOI:** 10.1155/2013/678063

**Published:** 2013-10-10

**Authors:** Luc Sensebé, Sandrine Fleury-Cappellesso

**Affiliations:** ^1^UMR5273 CNRS, UPS, EFS—INSERM U1031, STROMALab, Toulouse, France; ^2^EFS Pyrénées-Méditerranée, Toulouse, France

## Abstract

Due to their multi/pluripotency and immunosuppressive properties, mesenchymal stem/stromal cells (MSCs) are important tools for treatment of immune disorders and tissue repair. The increasing uses of MSCs lead to the development of production processes that need to be in accordance with good manufacturing practices (GMP). In Europe, MSCs are somatic cell-therapy products, referred to as advanced-therapy medicinal products (ATMPs), and in the United States MSCs must comply with current good tissue practice requirements. The safety and efficacy of MSCs must be ensured, whatever the cell source, and studies of dose and biodistribution are important aspects of safety testing. Preclinical data on biodistribution and pharmacodynamics are mandatory for approval. It is important to demonstrate that MSCs do not have unwanted homing that could drive to inappropriate differentiation in some organ or to support cancer development as suggested in some experiments. All these aspects should be addressed in a risk-based approach according to recently published guidelines by EMA. In the present article, we summarize the main approaches for labeling and tracking of infused MSCs, report on current animal models, and give an overview of available results on biodistribution.

## 1. Introduction

One of the most promising tools in cellular therapy and regenerative medicine is the use of mesenchymal stem/stromal cells (MSCs) because of their dual differentiation potential and immune regulatory properties. First described by Alexander Friedenstein, in the 1960s/70s [1], as mesodermic-derived nonhematopoietic bone marrow (BM) cells adhering to plastic that developed into colonies with a fibroblastic appearance, MSCs were called stem cells of skeletal tissues (e.g., bone, cartilage) [2]. The first clinical use was their combination with biomaterials to repair long bone fractures [3]. Later, emerging cells of the MSC type were found to originate from the neural crest and not the mesoderm [4], and cells with some features of BM-MSCs were found in almost all tissues of the fetus, neonate, and adult [5].

An international expert panel [6] described the common minimal criteria for cells in the MSC category: cells adhering to plastic; able to form colony-forming unit fibroblasts (CFU-Fs); positive for membrane markers CD90, CD73, and CD105 but negative for the hematopoietic molecules CD45, CD34, and HLA-DR; and able to differentiate via osteoblastic, chondrocytic, and adipocytic pathways. These main characteristics apply to cultured BM-MSCs, but some differences appear depending on the tissue of origin (e.g., adipose tissue-derived MSC expression of CD34 and CD54 [7]). As well, MSCs from different tissues are not equivalent in phenotype and function [8].

The MSC field has moved rapidly with the demonstration that *ex vivo*-expanded MSCs show immuno suppressive properties that were exploited in a wide range of phase 2 clinical trials from treatment of drug-resistant graft-versus-host disease [9] to organ transplantation [10]. Finally, MSCs have trophic effects mediated by numerous growth factors and the cytokines they produce [11].

Cells referred to as MSCs originating from various tissues are now widely used in clinical trials. In Europe, MSCs are somatic cell-therapy products, referred to as advanced-therapy medicinal products (ATMPs) and are under European Regulation No. 1394/2007. In the United States, like the production of any other cellular and tissue-based product, MSCs must comply with Current Good Tissue Practice requirements, under the US Code of Federal Regulations. The safety and efficacy of MSCs must be ensured, whatever the cell source, and studies of dose and biodistribution are important aspects of safety testing. Moreover, before approval, regulatory authorities require preclinical data on bio-distribution and pharmacodynamics; in the European Union, these data should be generated according to the European Pharmacopoeia. In fact, biodistribution of MSCs is one of the main preclinical data required for safety; it is important to demonstrate that MSCs do not have unwanted homing that could drive to inappropriate differentiation in some organ [12] or support cancer development as suggested in some experiments [13, 14]. All these aspects should be addressed in a risk-based approach. Guideline on the risk-based approach was recently published by the Committee for Advanced Therapies (CAT) of EMA (Guideline on the risk-based approach according to annex I, part IV of directive 2001/83/EC for ATMPs: EMA/CAT/CPWP/686637/2011). The guideline defines risks, for example, unwanted targeting of cells/organs, unwanted tissue formation, and tumor formation; risk factors, as qualitative or quantitative characteristics that contribute to a specific risk following administration of ATMPs. All available information on risks and risk factors should be integrated for building risk profiling, and bio-distribution is one of the main preclinical data giving information in different risks and risk factors. To reach this target, biodistribution has to be conducted according to the European Pharmacopoeia and according to the Good Laboratory Practices.

After injection of MSCs, whatever the route, the study of biodistribution is challenging; different aspects must be analyzed, the main ones being methods for cell labeling or staining and tracking, animal models, and relevance for human use.

## 2. Labeling and Tracking of MSCs

As for other living cells, MSCs can be labeled or stained by numerous methods. They can be easily labeled with an intracellular dye such as 5-carboxyfluorescein diacetate succinimidyl ester or a synthetic nucleoside such as 5-bromo-2′-deoxyuridine (BrdU) during culture. BrdU is incorporated into the newly synthesized DNA of replicating cells, substituting for thymidine during DNA replication. During the labeling phase, this method requires dividing cells, which could result in nonhomogeneous labeling, with an inconsistent percentage of residual unlabeled MSCs. After infusion, labeled MSCs can be detected in tissues with BrdU-specific antibodies [15]. For these two labeling molecules, cell division results in sequential halving of incorporated molecules that could decrease the quantity per cell to below the detection threshold. It should be stressed that these methods are more useful for studying distribution or local spreading after intraorgan injection. Another easy method of cell labeling in MSCs is transfection of cells with a gene coding a fluorescent protein (e.g., green fluorescent protein [GFP]) [16]. Two problems can be encountered: autofluorescence of some tissues and nonspecific expression due to reuptake of protein by other cells such as macrophages. Finally, all these methods do not allow for *in vivo* followup of the biodistribution of MSCs; after animals are killed, specific staining and histological analyses of different tissues are required. For *in vivo* followup, several labeling techniques are useful: labeling with a radioactive tracer molecule such as technetium-99 m, ^111^indium-oxine [17] or fludeoxyglucose (18FDG), with positron emission tomography (PET) [18]; transfection of MSCs with luciferase and external camera-based acquisition of bioluminescence after injection of a substrate (luciferin) [19]; or incorporation of iron magnetic nanoparticles in MSCs and *in vivo* MRI [20, 21].

The use of different molecules for labeling may have consequences on MSC functions particularly in differentiation or immunosuppression. With iron nanoparticles, studies have shown contradictory results: Chang et al. [20] reported impaired *in vitro *osteogenic and chondrogenic differentiations after labeling of human MSCs with amine-surface-modified superparamagnetic iron oxide nanoparticles; on the contrary, Schmidtke-Schrezenmeier et al. [21] found that labeling MSCs with iron oxide-poly(L-lactide) nanoparticles did not affect a large set of functions of labelled MSCs: viability, phenotype, proliferation, differentiation, or immunosuppression. These conflicting results could be related to differences in iron particles used, number of particles per cell, or the production processes and age of cells.

Finally, in xenogeneic models, without previous labeling, human MSCs can be tracked by the use of quantitative PCR (qPCR) of human Alu sequences [22] or histology after labeling of human specific antigens. The human Alu sequences are highly repeated, species-specific, and 300 bp sequences. Because of their high repetition and species specificity, Alu sequences are a marker of choice to detect invasion of a few human cells in a mouse organ. The presence and quantification of the Alu marker is evaluated by qPCR amplification with DNA extracted from multiple tissue samples from mice as templates and from healthy noninduced mice as a negative control. A standard curve indicating the quantity of human DNA versus mouse DNA can be established with known amounts of DNA extracted from the different mouse tissues and human MSCs. This standard curve allows for estimating the number of cells per weight unit of tissue as 230 human cells/g of tissue (LS, personal data); with an improved technique, the threshold could reach 100 human cells/organ [23].

## 3. Animal Models

As for other preclinical data, the choice of animal model is critical. For studying biodistribution, a first step could be the use of MSCs from the same animal species. However, regulatory authorities require data for human MSCs. To prevent rejection of human MSCs, which could interfere with the biodistribution and maintenance within different tissues, the most popular models are injection in immunocompromised rodents, such as nude mice, or to prevent any rejection, NOD/SCID mice. Owing to the incomplete disappearance of immunity in nude and even NOD/SCID mice, the best model seems to be NOD-Rag mice [24]. The main problem with mouse models is the dose of intravenous-infused MSCs, generally between 0.5 to 2 million, to prevent death by lung embolization. These doses, corresponding to 100 to 200 million of MSCs/kg, deviate greatly from the clinically relevant doses for humans. In a European program of the 7th Framework Program of the European Commission (REBORNE: FP7-HEALTH-241879), we studied bio-distribution of MSCs from two different tissues: bone marrow and adipose tissue. Studies were conducted in SCID mice after IV infusion or after subcutaneous implantation of MSCs loaded in a ceramic scaffold. Whatever the sources of MSCs, using qPCR for human Alu sequences, there was never any unwanted homing of MSCs after IV infusion. At day 7, on the contrary of AT-MSC, we did not find any BM-MSCs in lungs; at day 91, only with some AT MSCs there was remaining human DNA in lungs. Following subcutaneous implantation of MSCs + scaffold, there were never any recirculation and homing in other organs. Moreover, histological analysis of different organs did not show any tumor formation. These data demonstrated the safety of using BM and AT MSCs, and based on these results and other preclinical data, the French, German, Spanish, and Italian regulatory authorities delivered authorization for starting clinical studies in bone repair.

Use of other models, such as nonhuman primates, seems more relevant but is difficult for ethical reasons, and when testing human MSCs, use of immuneosuppressive drugs is mandatory.

Regarding intended clinical uses, models should be relevant for the route of MSC infusion—intravenous, intra-arterial, or local injection or implantation—and for the treated condition. MSCs preferentially home to injured tissues such as radiation-injured tissues [25]. The bio-distribution and pharmacodynamics must be studied in both normal and animal models of diseases.

Finally, because MSCs from different tissues are not equivalent [8] and culture processes differ, MSCs from different types of preparation should be tested.

## 4. Biodistribution of MSCs

### 4.1. In Animal Models

Detection of the human Alu sequence revealed that human BM-MSCs intravenously injected in mice are rapidly trapped in lungs. After this first embolization, MSCs may be recirculated, as demonstrated 15 min postinfusion by culture of peripheral blood mononuclear cells showing CFU-Fs of human origin [23]. During a short followup, infused human MSCs home to different organs, mainly liver, but this secondary recirculation seems to concern only a small amount of infused MSCs. In this model, at 48 and 96 hr postinfusion, only 0.04% and 0.01% of injected human MSCs were recovered in 6 tissues (liver, spleen, pancreas, brain, kidney, heart, and bone marrow). On studying human BM-MSC distribution in mice for a longer time (7 days and 3 months), human cells were found only at 7 days in spleen (LS, personal data). Labeling techniques appear to be important: labeling with technetium-99 m and intravenous infusion of human BM-MSCs revealed a long-term persistence of human cells up to 4 to 13 months in bone, bone marrow, spleen, muscle, and cartilage [26].

BM-MSCs intravenously infused, as human AT-MSCs, adipose tissue are trapped first in lung. Human adipose tissue MSCs transfected with luciferase in nude mice showed that human cells were cleared at 1 week from the lung, and then persisted for 31 weeks in liver [19]. On detecting the Alu sequence of human adipose tissue MSCs in NOD/SCID mice, 7 days postinfusion, human cells were located in spleen but mainly remained within lungs and persisted there for 3 months without any other secondary localization (LS, personal data). Recently, after intravenous infusion of human adipose tissue MSCs, human cells were rapidly cleared and mostly found at days 11 and 28 in lungs and gastrointestinal tract. As well, results were highly variable: only 17% to 25% of mice were positive [22]. Regarding specific regulatory requirements, intravenously infused human MSCs were not found in testis or ovary [22, 27].

Findings were similar for human BM-MSCs intraarterially infused in mice and for intravenously infused cells [23] but differed by animal model used for the disease. For example, in rat with cerebral ischemia, injection of human BM-MSCs resulted in transient localization of human cells in the brain [28].

Results could differ when using animal MSCs in the same species for an abnormality. After intravenous infusion of ^111^indium-oxine-labeled rat MSCs, Yoon et al. observed brain uptake in rats with brain trauma [29]. In an irradiated primate model, intravenous infusion of primate GFP-labeled BM-MSCs and hematopoietic stem cells resulted in localization in bone marrow, skin, gut, and muscle from 12 to 82 days afterinfusion, with no cells in lungs [27].

Finally, in models with local injection, for example, intraarticular [22] and intramuscular [30], cells remained for a long time at the injection site. After intramuscular injection, no human DNA was detected in any evaluated tissues outside muscle [30]. In contrast, after intraarticular injection at different times (from day 11 to 186), human Alu sequences were found in heart, spleen, intestine, brain, testis, or liver of 10% to 20% of analyzed mice [22]. When cells are implanted with biomaterial or a scaffold, locally tracking and demonstrating persistence of human cells can be difficult [31], but BM-MSCs or AT-MSCs do not spread (LS, personal data).

### 4.2. In Humans

Few data are available on the bio-distribution of MSCs in humans. The results can be somewhat similar to these found in rodent models in terms of lung trapping, with differences in recirculation that could be related to the disease or species. Gholamrezanezhad et al. [17] used radiolabeled (^111^In-oxine) BM-MSCs intravenously infused in patients with advanced cirrhosis and found that after initial lung accumulation, BM-MSCs relocalized in liver and spleen: radioactivity decreased from 33.5% to 2% in lung and increased from 2% to 42% in spleen. In 3 patients receiving allogeneic BM-MSCs for treatment of corticoid-resistant graft-versus-host disease (GVHD) after hematopoietic stem cell transplantation, postmortem analysis of one patient revealed donor DNA targeting GVHD in a lymph node and in the gastrointestinal tract, but donor DNA was never found in lung, liver, or spleen [32].

## 5. Conclusions and Perspectives

Studies of MSC biodistribution are mandatory for safety reasons, regulatory requirements, and for risk-based approach of ATMPs uses. Labeling of MSCs is easy, but the limitations of each technique and the desired followup should be considered. Classical techniques (e.g., GFP labeling) do not allow for *in vivo* followup. A labeling system allowing for external followup (e.g., luciferase, iron particles, or radioactivity) may decrease detection sensitivity. Detection of human Alu sequences by qPCR appears to be simple and sensitive and can be used when continuous external followup is not required. In preclinical settings, the main animal models used are immunocompromised mice (nude, NOD/SCID, or NOG-Rag). Although easy to use and informative, such models have limitations: they are xenogeneic, human MSCs are larger than mouse MSCs, and the physiologic features differ between rodents and humans. Whatever the model, MSCs seem to be initially trapped in lungs. After this lung embolization, MSCs are recirculated, but the number of recirculating cells seems low, and secondary homing occurs at the liver, spleen, and inflammatory or injured sites.

Two main recommendations are (1) use of the most sensitive technique for labeling and/or tracking and (2) a relevant animal model in terms of immune rejection potential and the intended disease to treat. Finally, animal models are required, informative but not enough, and more data in humans are needed. It could be discussed with regulatory authorities performing some phase 1 studies for validating tracking systems and biodistribution in human. Moreover, registries reporting all available data in human will be of major importance.
